# Unofficinal Medicines

**Published:** 1867-06

**Authors:** 


UNOFFICINAL MEDICINES.

Dr. Hibbard called up his resolutions on unofficinal medicines, *viz.:*—

*Resolved*, That the habit of using unofficinal preparations of medicine by physicians, except where there is no officinal preparation that will answer the purpose as well, is unscientific and imprudent, tending to demoralize the therapeutist, and to encourage irregular pharmaceutists and nostrum makers, and should be abandoned.

*Resolved*, That the profession should not patronize druggists who are engaged in the manufacture of nostrums.

Dr. Hibbard explained that the object of the resolution was to discourage the use of medicines prepared in peculiar and attractive forms, and insidiously introduced throughout the country, that might be genuine and might not, but which, at any rate, tended to confuse the practice.

Dr. Post suggested that instead of the word "unofficinal," the word "secret" be used.

Dr. Levick spoke of these prepared medicines as becoming a great evil. No prescription should be written which could not be put up by any apothecary.

Dr. Cox expressed the opinion, that the profession should not patronize any druggist who manufactured, advertised, or sold quack medicines. Such druggists, while making enemies to the regular profession, taking their patients from them, had no right to approbation or support from those who confined themselves to regular and legitimate prescriptions.

Dr. Howard remarked:—We have a right to regulate our own affairs, but we have no right to interfere with the business of the apothecary, or hinder him from selling what he pleases.

Dr. H. A. Johnson, of Illinois, said:—The question seems to be whether it is or is not professional to use remedies not recognized in the standard pharmacopœia of the profession. It does not become this profession to countenance quackery, but we should be free to use any of those remedies presented by the so-called Eclectics, or Hydropathics, or resort to the use of preparations first advanced by Homœopathists, provided we find them useful in overcoming disease. We should stand on the broad platform of science; and no matter from what source remedies come, whether presented by a school of medicine called quackery, or through channels which we are accustomed to call legitimate, we should accept what proves to be useful. In the hands of scientific men they will become legitimate. And these preparations and elixirs that have been referred to, they are not secret remedies; we know what they are, and what their value, and I must feel at liberty to use them, get them where I can.

After further discussion, the resolutions were referred to the Committee on By-Laws.

